# Time to Continuous Renal Replacement Therapy Initiation and 90-Day Major Adverse Kidney Events in Children and Young Adults

**DOI:** 10.1001/jamanetworkopen.2023.49871

**Published:** 2024-01-02

**Authors:** Katja M. Gist, Shina Menon, Pilar Anton-Martin, Amee M. Bigelow, Gerard Cortina, Akash Deep, Sara De la Mata-Navazo, Ben Gelbart, Stephen Gorga, Isabella Guzzo, Kenneth E. Mah, Nicholas J. Ollberding, H. Stella Shin, Sameer Thadani, Amanda Uber, Huaiyu Zang, Michael Zappitelli, David T. Selewski

**Affiliations:** 1 Cincinnati Children’s Hospital Medical Center, University of Cincinnati College of Medicine, Cincinnati, Ohio; 2 Seattle Children’s Hospital, University of Washington, Seattle; 3 Children’s Hospital Philadelphia, Philadelphia, Pennsylvania; 4 Nationwide Children’s Hospital, The Ohio State University College of Medicine, Columbus; 5 Medical University of Innsbruck, Innsbruck, Austria; 6 King’s College Hospital, London, England; 7 Gregorio Marañón University Hospital; Gregorio Marañón Health Research Institute, Madrid, Spain; 8 Royal Children’s Hospital, University of Melbourne, Murdoch Children’s Research Institute, Melbourne, Victoria, Australia; 9 University of Michigan Medical School, C.S. Mott Children’s Hospital, Ann Arbor; 10 Bambino Gesù Children’s Hospital, IRCCS, Rome, Italy; 11 Stanford University School of Medicine, Palo Alto, California; 12 Children’s Healthcare of Atlanta, Emory University, Atlanta, Georgia; 13 Baylor College of Medicine, Texas Children’s Hospital, Houston; 14 University of Nebraska Medical Center, Children’s Hospital & Medical Center, Omaha; 15 University of Utah, Primary Children’s Hospital, Salt Lake City; 16 Hospital for Sick Children, Toronto, Ontario, Canada; 17 Medical University of South Carolina, Charleston

## Abstract

**Importance:**

In clinical trials, the early or accelerated continuous renal replacement therapy (CRRT) initiation strategy among adults with acute kidney injury or volume overload has not demonstrated a survival benefit. Whether the timing of initiation of CRRT is associated with outcomes among children and young adults is unknown.

**Objective:**

To determine whether timing of CRRT initiation, with and without consideration of volume overload (VO; <10% vs ≥10%), is associated with major adverse kidney events at 90 days (MAKE-90).

**Design, Setting, and Participants:**

This multinational retrospective cohort study was conducted using data from the Worldwide Exploration of Renal Replacement Outcome Collaborative in Kidney Disease (WE-ROCK) registry from 2015 to 2021. Participants included children and young adults (birth to 25 years) receiving CRRT for acute kidney injury or VO at 32 centers across 7 countries. Statistical analysis was performed from February to July 2023.

**Exposure:**

The primary exposure was time to CRRT initiation from intensive care unit admission.

**Main Outcomes and measures:**

The primary outcome was MAKE-90 (death, dialysis dependence, or persistent kidney dysfunction [>25% decline in estimated glomerular filtration rate from baseline]).

**Results:**

Data from 996 patients were entered into the registry. After exclusions (n = 27), 969 patients (440 [45.4%] female; 16 (1.9%) American Indian or Alaska Native, 40 (4.7%) Asian or Pacific Islander, 127 (14.9%) Black, 652 (76.4%) White, 18 (2.1%) more than 1 race; median [IQR] patient age, 8.8 [1.7-15.0] years) with data for the primary outcome (MAKE-90) were included. Median (IQR) time to CRRT initiation was 2 (1-6) days. MAKE-90 occurred in 630 patients (65.0%), of which 368 (58.4%) died. Among the 601 patients who survived, 262 (43.6%) had persistent kidney dysfunction. Of patients with persistent dysfunction, 91 (34.7%) were dependent on dialysis. Time to CRRT initiation was approximately 1 day longer among those with MAKE-90 (median [IQR], 3 [1-8] days vs 2 [1-4] days; *P* = .002). In the generalized propensity score–weighted regression, there were approximately 3% higher odds of MAKE-90 for each 1-day delay in CRRT initiation (odds ratio, 1.03 [95% CI, 1.02-1.04]).

**Conclusions and Relevance:**

In this cohort study of children and young adults receiving CRRT, longer time to CRRT initiation was associated with greater risk of MAKE-90 outcomes, in particular, mortality. These findings suggest that prospective multicenter studies are needed to further delineate the appropriate time to initiate CRRT and the interaction between CRRT initiation timing and VO to continue to improve survival and reduce morbidity in this population.

## Key Points

**Question:**

Is a longer time to continuous renal replacement therapy (CRRT) initiation associated with worse major adverse kidney events at 90 days (MAKE-90)?

**Findings:**

In this cohort study of 969 patients with data for the primary outcome of MAKE-90, a propensity score–weighted analysis found that each 1-day delay in CRRT initiation was associated with 3% higher odds of MAKE-90.

**Meaning:**

These results suggest that delaying initiation of CRRT in children who are critically ill was associated with an increased risk of adverse events, including death, dialysis dependence, and persistent kidney dysfunction at 90 days.

## Teaser

This cohort study investigates whether the timing of continuous renal replacement therapy (CRRT) initiation, with and without consideration of volume overload, is associated with major adverse kidney events at 90 days among children and young adults.

## Introduction

Acute kidney injury (AKI) and volume overload (VO) are common in children who are critically ill and at risk for increased morbidity and mortality.^1,2,3,4,5,6,7,8^ Continuous renal replacement therapy (CRRT) is used when medical therapy cannot maintain fluid balance, or complications of AKI develop.^9,10,11,12,13,14,15^ In children, there is substantial heterogeneity in CRRT practice patterns regarding initiation, dose, fluid removal, and anticoagulation.^16,17,18,19^ The Worldwide Exploration of Renal Replacement Outcomes Collaborative in Kidney Disease (WE-ROCK) is a multidisciplinary, international registry formed in 2021 to better understand practice patterns and outcomes of CRRT in a contemporary cohort of children who are critically ill.^20^

Over the last decade, attention has focused on determining the optimal timing for initiating CRRT. Determining the optimal timing of CRRT initiation remains a top research priority in the most recent Kidney Disease: Improving Global Outcomes (KDIGO) Conference on Controversies in AKI^21,22^ and the pediatric Acute Disease Quality Initiative Consensus statements.^23^ Multiple randomized trials in adults evaluating the effect of timing of initiation on CRRT outcomes have yielded conflicting results.^14,15,24,25,26,27,28,29^ In children, there are no randomized trials evaluating the timing of CRRT initiation and outcomes, and most studies are small, single center in design with varied timing definitions.^11,12,30,31,32,33^ Therefore, a similar controversy as to the optimal timing of CRRT initiation in children exists. This represents a substantial gap in our knowledge and opportunity to improve outcomes in pediatric critical care nephrology.

This cohort study is a planned analysis of the WE-ROCK registry focused on evaluating the association of timing of CRRT initiation and VO at CRRT initiation with outcomes. The study aims to (1) describe the demographic and clinical characteristics of early (≤2 days) vs late (>2 days) CRRT initiation, (2) describe the differences in outcomes among patients stratified by time to CRRT initiation, and (3) evaluate the interaction of CRRT initiation timing and VO with outcomes (<10% and ≥10%). We hypothesized that longer time to CRRT initiation and greater VO at CRRT initiation would be associated with worse outcomes.

## Methods

The WE-ROCK registry is a retrospective multicenter study of children and young adults, aged birth to 25 years, receiving CRRT for AKI and VO from 2015 to 2021 from 32 centers across 7 countries (United States of America, Canada, United Kingdom, Italy, Spain, Austria, and Australia). Each center obtained study approval from their institutional review boards, and informed consent was waived because of the retrospective nature of the study and inability to obtain data on all eligible patients for improved generalizability. This study follows the Strengthening the Reporting of Observational Studies in Epidemiology (STROBE) reporting guideline (eTable 1 in Supplement 1).^34^ Patients with a history of end-stage kidney disease (ESKD), extracorporeal membrane oxygenation, peritoneal dialysis prior to CRRT, treated with CARPEDIEM (agreement with another registry), or CRRT for a non-AKI/VO indication (ingestion, inborn error of metabolism, hyperammonemia) were excluded from the registry.

A detailed description of WE-ROCK and its data collection have been previously reported.^20^ Baseline kidney function was determined using the serum creatinine in the 90 days prior to admission. For those without a baseline serum creatinine, it was calculated using the bedside Schwartz equation assuming an estimated glomerular filtration rate of 100 mL/min/1.73m^2^.^35,36^ We used weight to define VO when available based on recent consensuses guidelines.^23,37,38^ Percentage VO was calculated from intensive care unit (ICU) admission to CRRT initiation using the following equation as previously described:^39^ percentage VO = [(daily weight − ICU admission weight) / (ICU admission weight)] × 100. Race and ethnicity data were collected and reported because of the potential for differences in outcomes.

The primary variable was time to CRRT initiation anchored to ICU admission, defined as days from ICU admission to CRRT initiation. ICU admission was used as the anchor to make comparisons to existing studies.^19,31,33,40^ We categorized time to CRRT initiation as a binary variable based on the cohort’s median time to CRRT (early: ≤2 days; late: >2 days). This binary timing variable was combined with an a priori–identified VO metric (<10% and ≥10% VO)^41^ (eFigure 1 in Supplement 1).

The primary outcome was major adverse kidney events at 90 days (MAKE-90), defined as death or persistent kidney dysfunction (dialysis dependence or >25% decline in estimated glomerular filtration rate from baseline) and 90-day mortality alone. Secondary outcomes included: (1) ventilator-free days and (2) ICU-free days assessed during the first 28 ICU days. Ventilator-free and ICU-free days are described as the number of days, out of a maximum of 28, for which the patient did not receive ventilation or was not in the ICU. The number of ventilator-free and ICU-free days for patients who died was 0.^42^

### Statistical Analysis

Continuous variables were reported as median with IQR and were compared using Wilcoxon rank-sum tests or Kruskal-Wallis tests as appropriate. Categorical variables were reported as proportion with percentage and were compared using χ^2^ tests. A Kaplan-Meier curve was generated to depict the cumulative probability of receiving CRRT. We used the inverse probability of treatment weighting (IPTW) method using a covariate balancing generalized propensity score (CBGPS) to evaluate the association of CRRT initiation timing with outcomes.^43^ The weights were estimated for the continuous exposure using the CBGPS method (eMethods in Supplement 1)^43,44^ A candidate list of clinically relevant covariates (eFigure 2 in Supplement 1) included in the CBGPS model was determined a priori. The CBGPS model handled missing covariate data by incorporating their missing indicators in the weight estimation process. Extreme weights (eg, >10) were truncated and replaced with a weight of 10. The correlation between the continuous exposure and a given covariate was used to examine the balance assessment, with the value of less than 0.1 suggesting minimal confounding effect.^45^ A weighted univariate regression model in which the outcome was regressed on time to CRRT initiation as the sole primary variable and incorporating the CBGPS-based weights was used to estimate the exposure-outcome associations.

The weighted logistic regression models were used to estimate odds ratios (ORs) and 95% CIs for MAKE-90 and mortality at 90 days. Weighted ordinal regression models were used to fit 28-day ventilator-free days and ICU-free days. The ordinal regression model was used as it is advantageous for skewed outcome variables while preserving power.^46^ Fitted regression models after incorporating the inverse CBGPS weights included time to CRRT initiation as the sole primary variable. Standard errors were obtained using the Huber-White method to correct for the clustering of patients within hospitals. Outcomes of ventilator-free and ICU-free days were estimated via the ordinal regression model; a common OR and 95% CI was calculated, where an OR of less than 1 indicated a factor was associated with a worse clinical outcome (ie, increased health care utilization).

For the subphenotype analysis, similar statistical modeling methods were used. The model covariates included timing to CRRT initiation (early: ≤2 days vs late: >2 days), VO at CRRT initiation (<10% vs ≥10%), an interaction term between CRRT initiation and VO, comorbidity categories, sepsis at ICU admission, vasoactive inotrope score at CRRT initiation, and CRRT duration (days). The adjusted odds ratios (aORs) were obtained for each outcome with 95% CI. For continuous covariates, interquartile odds ratios, comparing 75th percentile to the 25th percentile were also reported. Wald χ^2^ test was used to assess the significance of the interaction term. Two-sided *P* < .05 was considered statistically significant. The multivariable results for the subphenotype analysis using the complete data were reported, and patients with missing data were excluded from these analyses. All statistical analyses were performed from February to July 2023 using R version 4.1.0 (R Project for Statistical Computing).^49^ The package WeightIt (version 0.13.1)^48^ was used for CBGPS weighting method. The logistic regression and ordinal regression models were fit using the lrm function in the rms package (version 6.0.0).^47^ The robust Huber-White variance was calculated using the robcov function in the rms package.

## Results

### Patient Characteristics

The WE-ROCK registry includes 996 patients. After exclusions (n = 27), 969 patients with data for the primary outcome (MAKE-90) remained (64 from 2015-2018 and 905 from 2018-2021) (eFigure 3 in Supplement 1). Among these 969 patients, 440 (45.4%) were female; 16 (1.9%) were American Indian or Alaska Native, 40 (4.7%) were Asian or Pacific Islander, 127 (14.9%) were Black, 652 (76.4%) were White, and 18 (2.1%) were more than 1 race; the median (IQR) patient age was 8.8 (1.7-15.0) years (Table 1). The most common admission category was shock/infection/trauma (360 patients [37.2%]), and 117 (12.1%) had 2 or more comorbidities. Baseline kidney function was measured for 541 patients (55.8%) and was a median (IQR) of 0.4 (0.22-0.62) mg/dL. The median (IQR) ventilator-free days was 12 (0-28) and median (IQR) ICU-free days was 0 (0-0).

**Table 1.** Demographics, Clinical Characteristics, and Outcomes of Patients With and Without MAKE-90^a^

Variable	Overall (N = 969)	No MAKE-90 (n = 339)	MAKE-90 (n = 630)	*P* value
Age, median (IQR), y	8.8 (1.7-15.0)	6.8 (2.0-14.0)	9.3 (1.4-15.6)	.32
Admission weight, median (IQR), kg	26.8 (11.6-54.9)	25.4 (12.1-55.5)	27.8 (11.0-54.4)	.51
Sex				
Female	440 (45.4)	149 (44.0)	291 (46.2)	.55
Male	529 (54.6)	190 (56.0)	339 (53.8)	
Race				
American Indian or Alaska Native	16 (1.9)	3 (1.0)	13 (2.4)	.53
Asian or Pacific Islander	40 (4.7)	15 (4.9)	25 (4.6)	
Black	127 (14.9)	51 (16.6)	76 (13.9)	
White	652 (76.4)	232 (76.9)	420 (76.9)	
More than 1 race	18 (2.1)	6 (2.0)	12 (2.2)	
Missing	116	32	84	
Ethnicity				
Hispanic or Latino	160 (18.6)	48 (15.9)	112 (20.1)	.16
non–Hispanic or Latino	700 (81.4)	254 (84.1)	446 (79.9)	
Missing	109	37	45	
Admit category				
Shock/infection/trauma	360 (37.2)	154 (45.4)	206 (32.7)	<.001
Respiratory failure	193 (19.9)	33 (9.7)	160 (25.4)	
Postsurgical/minor trauma	48 (5.0)	19 (5.6)	29 (4.6)	
CNS dysfunction	37 (3.8)	10 (2.9)	27 (4.3)	
Pain/sedation	8 (0.8)	3 (0.9)	5 (0.8)	
Primary cardiac disease	31 (3.2)	6 (1.8)	25 (4.0)	
Post cardiac surgery	49 (5.1)	18 (5.3)	31 (4.9)	
Heart failure/myopathy	39 (4.0)	12 (3.5)	27 (4.3)	
Other	204 (21.1)	84 (24.8)	120 (19.0)	
Comorbidity				
None	191 (19.7)	108 (31.9)	83 (13.2)	<.001
Respiratory	133 (13.7)	41 (12.1)	92 (14.6)	.32
Cardiac	192 (19.8)	47 (13.9)	145 (23.0)	<.001
Neurologic/neuromuscular	131 (13.5)	43 (12.7)	88 (14.0)	.65
Nephrologic/Urologic	91 (9.4)	26 (7.7)	65 (10.3)	.22
Hematologic	124 (12.8)	37 (10.9)	87 (13.8)	.24
Oncologic	220 (22.7)	59 (17.4)	161 (25.6)	.005
Immunologic	153 (15.8)	30 (8.8)	123 (19.5)	<.001
Gastrointestinal	184 (19.0)	63 (18.6)	121 (19.2)	.88
Endocrinologic	62 (6.4)	24 (7.1)	38 (6.0)	.62
Comorbidities, No.				
0	191 (19.7)	108 (31.9)	83 (13.2)	<.001
1	471 (48.6)	148 (43.7)	323 (51.3)	
2	190 (19.6)	52 (15.3)	138 (21.9)	
>2	117 (12.1)	31 (9.1)	86 (13.7)	
Baseline measured serum creatinine, median (IQR), mg/dL	0.40 (0.22-0.62) [n = 541]	0.5 (0.3-0.7) [n = 146]	0.37 (0.2-0.6) [n = 146]	<.001
Sepsis at ICU admission	442 (45.6)	143 (42.2)	299 (47.5)	.13
PRISM-III score at ICU admission, median (IQR)	14 (10-18)	14 (10-18)	14 (10-18)	.47
PELOD-2 score at CRRT initiation, median (IQR)	7 (4-9)	6 (4-8)	7 (5-10)	<.001
VIS score at CRRT initiation, median (IQR)	5 (0-20)	3 (0-15)	5 (0-20)	.01
VO at CRRT initiation, median (IQR), %	7.4 (2.4-18.1)	7.2 (2.2-16.2)	7.7 (2.5-19.9)	.20
Indexed UOP 24 h prior to CRRT Initiation, median (IQR), mL/kg/h	0.5 (0.1-1.2)	0.5 (0.2-1.4)	0.5 (0.1-1.2)	.14
Time to CRRT Initiation, median (IQR), d	2 (1-6)	2 (1-4)	3 (1-8)	.002
CRRT duration, median (IQR), d	6 (3-14)	5 (3-10)	8 (3-18)	<.001
Ventilator-free days	12 (0-28)	26 (16-28)	0 (0-21)	<.001
ICU-free days	0 (0-0)	0 (0-3)	0 (0-0)	<.001

Abbreviations: CNS, central nervous system; CRRT, continuous renal replacement therapy; ICU, intensive care unit; MAKE-90, major adverse kidney events at 90 days (persistent kidney dysfunction, dialysis dependence, or death); PELOD-2, pediatric logistic organ dysfunction score; PRISM-III, pediatric risk of mortality score III; UOP, urine output; VIS, vasoactive inotrope score; VO, percentage volume overload.

SI conversion factor: To convert creatinine to μmol/L, multiply by 88.4.

^a^ Categorical variables are presented as frequency with percentage. Continuous variables are presented as median with IQR. *P* values are calculated using χ^2^ test or Wilcoxon rank-sum test.

### Timing of CRRT Initiation

The median (IQR) time to CRRT initiation was 2 (1-6) days. A Kaplan-Meier curve for the time to CRRT initiation is presented in eFigure 4 in Supplement 1. Median (IQR) CRRT duration was 6 (3-14) days. There were 514 patients (52.5%) who initiated CRRT within 2 or fewer days of ICU admission. The characteristics of patients comparing early to late CRRT initiation are summarized in Table 2. Those who initiated CRRT more than 2 days after ICU admission differed significantly by admission category (more likely to have a respiratory or cardiac comorbidity), had lower Pediatric Risk of Mortality III (PRISM-III) score, and higher urine output prior to CRRT initiation.

**Table 2.** Demographics, Clinical Characteristics, and Outcomes of Patients With Early and Late Start of CRRT^a^

Variable	Patients, No. (%)			*P* value^b^
	Overall (N = 979)	Early CRRT (n = 514)	Late CRRT (n = 465)	
Age, y	8.8 (1.6-15.0)	9.0 (1.9-15.0)	8.2 (1.35-14.93)	.58
Admission weight, median (IQR), kg	26.8 (11.6-54.92)	27.0 (12.0-58.3)	26.5 (10.29-52.0)	.08
Sex				
Female	445 (45.5)	229 (44.6)	216 (46.5)	.59
Male	534 (54.5)	285 (55.4)	249 (53.5)	
Race				
American Indian or Alaska Native	16 (1.9)	9 (2.0)	7 (1.7)	.23
Asian or Pacific Islander	43 (5.0)	29 (6.5)	14 (3.4)	
Black	126 (14.6)	65 (14.5)	61 (14.7)	
White	660 (76.5)	339 (75.5)	321 (77.5)	
More than 1 race	18 (2.1)	7 (1.6)	11 (2.7)	
Missing	116	65	51	
Ethnicity				
Hispanic or Latino	160 (18.5)	77 (17.3)	83 (19.8)	.39
non–Hispanic or Latino	706 (81.5)	369 (82.7)	337 (80.2)	
Missing	113	68	45	
Admit category				
Shock/infection/trauma	364 (37.2)	208 (40.5)	156 (33.5)	<.001
Respiratory failure	194 (19.8)	87 (16.9)	107 (23.0)	
Postsurgical/minor trauma	49 (5.0)	24 (4.7)	25 (5.4)	
CNS dysfunction	39 (4.0)	25 (4.9)	14 (3.0)	
Pain/sedation	8 (0.8)	3 (0.6)	5 (1.1)	
Primary cardiac disease	31 (3.2)	4 (0.8)	27 (5.8)	
Post cardiac surgery	49 (5.0)	14 (2.7)	35 (7.5)	
Heart failure/myopathy	39 (4.0)	18 (3.5)	21 (4.5)	
Other	206 (21.0)	131 (25.5)	75 (16.1)	
Comorbidity				
None	193 (19.6)	119 (23.0)	74 (15.9)	.006
Respiratory	132 (13.5)	55 (10.7)	77 (16.6)	.01
Cardiac	191 (19.5)	60 (11.7)	131 (28.2)	<.001
Neurologic/neuromuscular	132 (13.5)	66 (12.8)	66 (14.2)	.6
Nephrologic/urologic	90 (9.2)	50 (9.7)	40 (8.6)	.62
Hematologic	132 (13.5)	73 (14.2)	59 (12.7)	.55
Oncologic	222 (22.7)	121 (23.5)	101 (21.7)	.55
Immunologic	153 (15.6)	87 (16.9)	66 (14.2)	.28
Gastrointestinal	185 (18.9)	92 (17.9)	93 (20.0)	.45
Endocrinologic	62 (6.3)	31 (6.0)	31 (6.7)	.78
Comorbidities, No.				
0	193 (19.6)	119 (23.0)	74 (15.9)	.003
1	479 (48.9)	246 (47.9)	233 (50.1)	
2	192 (29.6)	104 (20.2)	88 (18.9)	
>2	116 (1.8)	46 (8.9)	70 (15.1)	
Baseline measured serum creatinine, median (IQR), mg/dL	0.4 (0.22-0.62) [n = 540]	0.45 (0.25-0.69) [n = 254]	0.37 (0.20-0.60) [n = 285]	.006
Sepsis at ICU admission	446 (45.6)	228 (44.4)	218 (46.9)	.47
PRISM-III score at ICU admission, median (IQR)	14 (10-18)	15.00 (10.00-19.00)	13.00 (9.00-18.00)	.004
PELOD-2 score at CRRT initiation, median (IQR)	7 (4-9)	7 (4-9)	7 (5-9)	.21
VIS score at CRRT initiation, median (IQR)	5 (0-20)	5 (0-24)	5 (0-15)	.40
VO at CRRT initiation, median (IQR), %	7.4 (2.4-18.1)	4.8 (1.1-10.4)	12.5 (5.3-28.7)	<.001
Indexed UOP 24 h prior to CRRT initiation, median (IQR), mL/kg/h	0.5 (0.1-1.2)	0.3 (0.1-1.0)	0.6 (0.2-1.34)	<.001
CRRT duration, d	6 (3-14)	5 (2-12)	7 (4-17)	<.001
Ventilator-free days	13 (0-28)	19 (0-28)	0 (0-25)	<.001
ICU-free days	0 (0-0)	2 (0-16)	0 (0-0)	<.001
90-d mortality	367 (37.5)	173 (33.7)	194 (41.7)	.01
MAKE-90	629 (65.0)	314 (62.1)	315 (68.2)	.05

Abbreviations: CNS, central nervous system; CRRT, continuous renal replacement therapy; ICU, intensive care unit; MAKE-90, major adverse kidney events at 90 days (persistent kidney dysfunction, dialysis dependence, or death); PELOD-2, pediatric logistic organ dysfunction score; PRISM-III, pediatric risk of mortality score III; UOP, urine output; VIS, vasoactive inotrope score; VO, percentage volume overload.

SI conversion factor: To convert creatinine to μmol/L, multiply by 88.4.

^a^ Overall N for this table is 979, representing all eligible patients from which time to CRRT initiation was known. Categorical variables are presented as frequency with percent. Continuous variables are presented as median with IQR. *P* values are calculated using χ^2^ test or Wilcoxon rank-sum test.

Among those with late CRRT initiation, 315 of 465 patients (67.7%) had MAKE-90 vs 314 of 514 (61.1%) of those with early initiation (*P* = .054). Mortality at 90 days was significantly higher in those with late initiation (194 of 456 patients [42.5%] vs 173 of 514 patients [33.7%]; *P* = .01). Median (IQR) ventilator-free days (19 [0-28] days vs 0 [0-25] days; *P* < .001) and ICU-free days (2 [0-16] days vs 0 [0-0] days; *P* < .001) were also fewer among those with late initiation.

### VO at CRRT Initiation

The median (IQR) VO at CRRT initiation was 7.4% (2.4%-18.1%). Demographics and clinical characteristics differed among those with less than 10% VO vs those with at least 10% VO (eTable 2 in Supplement 1). Unadjusted median (IQR) VO at CRRT initiation for those who started CRRT at more than 2 days was 12.5% (5.3%-28.7%), and it was significantly higher than those who started CRRT at less than 2 days (4.8% [1.1%-10.4%]) (*P* < .001).

### Outcomes

MAKE-90 occurred in 630 patients (65.0%), of which 368 patients (58.4%) died. Among the 601 patients who survived, 262 (43.6%) had persistent kidney dysfunction (91 [34.7%] with dialysis dependence). Table 1 describes the demographics and clinical characteristics dichotomized by MAKE-90. The median (IQR) time to CRRT initiation was significantly longer among those with MAKE-90 (3 [1-8] days vs 2 [1-4] days; *P* = .002). CRRT duration was approximately 3 days longer among those with MAKE-90 (median [IQR] 8 [3-18] days vs 5 [3-10] days; *P* < .001).

### Associations With MAKE-90

The CBGPS weighted regression model for MAKE-90 found that for each day of waiting to initiate CRRT, there were 3% greater odds of MAKE-90 (OR, 1.03 [95% CI, 1.02-1.04]). There were 21% greater odds of MAKE-90 in patients with CRRT initiated at ICU day 6 compared with those with CRRT initiation on ICU day 1 (OR, 1.21 [95% CI, 1.16-1.26]) (Figure A). The conventional multivariable outcome regression model with adjustment for the same covariates in the generalized propensity score model was used to confirm the estimates obtained by CBGPS weighted regression (eTable 3 in Supplement 1).

**Figure.**
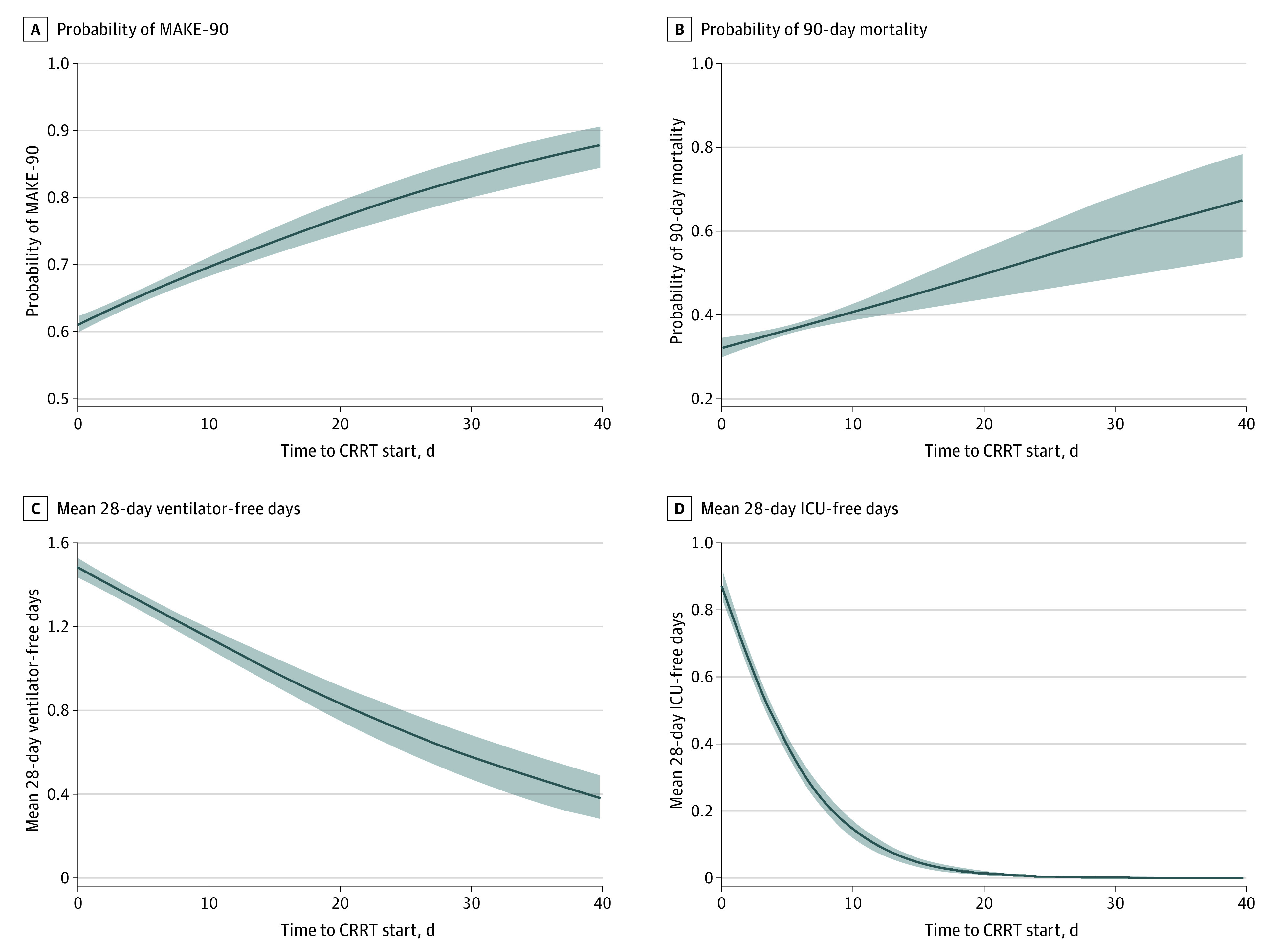
Projections of Major Adverse Kidney Events at 90 Days (MAKE-90), Mortality, Ventilator-Free Days, and Intensive Care Unit (ICU)–Free Days The figure shows projections of MAKE-90, mortality, ventilator-free days, and ICU-free days as a function of time to continuous renal replacement therapy (CRRT) initiation (days) from the weighted logistic regression and weighted ordinal regression model by generalized propensity score. The probability of MAKE-90 increased with longer time to CRRT initiation (A). Similarly, the probability of 90-day mortality also increased with longer time to CRRT initiation (B). Out of a maximum of 28 days, the mean number of ventilator-free days, where death acts as a competing risk defaulting to 0 days, decreased with longer time to CRRT initiation (C). The mean number of ICU-free days out of a maximum of 28 days also decreased with longer time to CRRT initiation (D).

### Associations With Other Outcomes

There were 4% greater odds of 90-day mortality for every 1-day delay in initiation (OR, 1.04 [95% CI, 1.02-1.06]). There were 20% greater odds of 90-day mortality for CRRT initiated at 6 days from ICU admission compared with those initiated at 1 day (OR, 1.20 [95% CI, 1.11-1.31]) (Figure B). Patients who initiated CRRT on ICU day 6 had significantly fewer ventilation-free days (OR, 0.78 [95% CI, 0.72-0.84]) (Figure C) and significantly fewer ICU-free days (OR, 0.31 [95% CI, 0.29-0.33]) (Figure D) as compared with those who initiated CRRT on ICU day 1.

### VO at CRRT Initiation and Outcomes

eTable 2 in Supplement 1 describes the association of VO greater than or equal to 10% with outcomes. In bivariable analysis, there were significant differences between VO less than 10% vs VO greater than or equal to 10% at CRRT initiation and (1) median (IQR) ventilator-free days (17 [0-28] days vs 3 [0-28] days; *P* < .001) and (2) median (IQR) ICU-free days (0 ([0-15] days vs 0 [0-2] days; *P* < .001). VO greater than 10% at CRRT initiation was not associated with MAKE-90 or mortality.

### CRRT Timing and VO Subphenotypes

Demographics, clinical characteristics, and outcomes of each of the 4 subphenotypes are summarized in Table 3. MAKE-90 and 90-day mortality were lowest in the early CRRT initiation subphenotype. Mortality was significantly higher among the CRRT at greater than 2 days and VO less than 10% subphenotype (85 patients [44.0%]) (*P* < .001). There were also significant differences in hospital-free days across the 4 subphenotypes.

**Table 3.** Demographics, Clinical Characteristics, and Outcomes of Time to CRRT Initiation and VO Subphenotypes^a^

Variable	Early CRRT^b^		Late CRRT^c^		*P* value^d^
	<10% VO (n = 378)	≥10% VO (n = 136)	<10% VO (n = 193)	≥10% VO (n = 268)	
Age, median (IQR), y	10.6 (2.8-16.0)	3.6 (0.9-10.6)	13.0 (5.7-16.6)	3.7 (0.8-12.9)	<.001
Admission weight, median (IQR), kg	37.7 (14.1-63.0)	15.3 (9.3-33.4)	46.7 (22.5-67.4)	16.1 (8.3-36.7)	<.001
Sex					
Female	173 (45.8)	56 (41.2)	87 (45.1)	128 (47.8)	.66
Male	205 (54.2)	80 (58.8)	106 (54.9)	140 (52.2)	
Race					
American Indian or Alaska Native	8 (2.4)	1 (0.8)	2 (1.1)	5 (2.1)	.47
Asian or Pacific Islander	22 (6.7)	7 (5.9)	9 (5.1)	5 (2.1)	
Black	44 (13.3)	21 (17.6)	24 (13.7)	37 (15.7)	
White	250 (75.8)	89 (74.8)	134 (76.6)	183 (77.9)	
More than 1 race	6 (1.8)	1 (0.8)	6 (3.4)	5 (2.1)	
Missing	48	17	18	33	
Ethnicity					
Hispanic or Latino	68 (20.5)	9 (7.8)	42 (24.3)	40 (16.5)	.003
non–Hispanic or Latino	263 (79.5)	106 (92.2)	131 (75.7)	203 (83.5)	
Missing	47	21	20	25	
Admit category					
Shock/infection/trauma	129 (34.1)	79 (58.1)	50 (25.9)	106 (39.6)	<.001
Respiratory failure	70 (18.5)	17 (12.5)	46 (23.8)	61 (22.8)	
Postsurgical/minor trauma	16 (4.2)	8 (5.9)	6 (3.1)	19 (7.1)	
CNS dysfunction	21 (5.6)	4 (2.9)	9 (4.7)	5 (1.9)	
Pain/sedation	2 (0.5)	1 (0.7)	2 (1.0)	3 (1.1)	
Primary cardiac disease	3 (0.8)	1 (0.7)	8 (4.1)	18 (6.7)	
Post cardiac surgery	11 (2.9)	3 (2.2)	10 (5.2)	23 (8.6)	
Heart failure/myopathy	16 (4.2)	2 (1.5)	16 (8.3)	5 (1.9)	
Other	110 (29.1)	21 (15.4)	46 (23.8)	28 (10.4)	
Comorbidity					
None	87 (23.0)	31 (22.8)	28 (14.5)	45 (16.8)	.04
Respiratory	39 (10.3)	16 (11.8)	28 (14.5)	48 (17.9)	.04
Cardiac	42 (11.1)	18 (13.2)	51 (26.4)	79 (29.5)	<.001
Neurologic/neuromuscular	39 (10.3)	27 (19.9)	25 (13.0)	40 (14.9)	.03
Nephrologic/urologic	38 (10.1)	12 (8.8)	20 (10.4)	19 (7.1)	.55
Hematologic	52 (13.8)	21 (15.4)	32 (16.6)	27 (10.1)	.19
Oncologic	91 (24.1)	30 (22.1)	50 (25.9)	50 (18.7)	.25
Immunologic	67 (17.7)	20 (14.7)	31 (16.1)	35 (13.1)	.44
Gastrointestinal	67 (17.7)	25 (18.4)	30 (15.5)	62 (23.2)	.18
Endocrinologic	17 (4.5)	14 (10.3)	14 (7.3)	17 (6.3)	.11
Comorbidities, No.					
0	87 (23.0)	31 (22.8)	28 (15.5)	45 (16.8)	.03
1	183 (48.4)	63 (46.3)	100 (51.8)	132 (49.3)	
2	80 (21.2)	24 (17.6)	36 (18.7)	51 (19.0)	
>2	28 (7.4)	18 (13.2)	29 (15.0)	40 (14.9)	
Baseline measured serum creatinine, median (IQR), mg/dL	0.5 (0.3-0.7) [n = 189]	0.3 (0.2-0.7) [n = 65]	0.5 (0.3-0.7) [n = 116]	0.3 (0.2-0.5) [n = 116]	<.001
Sepsis at ICU admission	146 (38.6)	82 (60.3)	83 (43.0)	135 (50.4)	<.001
PRISM-III score at ICU admission, median (IQR)	14 (10-18)	16 (12-21)	12 (9-17)	14 (9-18)	<.001
PELOD-2 score at CRRT initiation, median (IQR)	6 (3-9)	8 (6-11)	6 (4-8)	7 (5-10)	<.001
VIS score at CRRT initiation, median (IQR)	0 (0-15)	19 (9-39)	3 (0-12)	5 (0-17)	<.001
VO at CRRT initiation, median (IQR), %	2.64 (0.51-5.72)	15.09 (12.07-24.61)	3.78 (0.93-6.96)	26.23 (16.57-41.58)	<.001
Indexed UOP 24 h prior to CRRT initiation, median (IQR), mL/kg/h	0.3 (0.16-0.9)	0.4 (0.1-1.1)	0.6 (0.2-1.3)	0.7 (0.3-1.4)	<.001
CRRT duration, d	5 (2-12)	7 (3-13)	7 (3-16)	7 (4-17)	<.001
ICU mortality	113 (30)	51 (38)	78 (40)	107 (40)	.02
90-day mortality	117 (31)	56 (41)	85 (44)	108 (40)	.007
MAKE-90	231 (62)	83 (61)	130 (67)	182 (69)	.26

Abbreviations: CNS, central nervous system; CRRT, continuous renal replacement therapy; ICU, intensive care unit; MAKE-90, major adverse kidney events at 90 days (persistent kidney dysfunction, dialysis dependence, or death); PELOD-2, pediatric logistic organ dysfunction score; PRISM-III, pediatric risk of mortality score III; UOP, urine output; VIS, vasoactive inotrope score; VO, percentage volume overload.

SI conversion factor: To convert creatinine to μmol/L, multiply by 88.4.

^a^ There were 975 patients included in this analysis (5 excluded due to missing percentage VO). Categorical variables are presented as frequency with percentage.

^b^ Early CRRT defined as initiation less than or equal to 2 days after ICU admission.

^c^ Late CRRT defined as initiation greater than 2 days after ICU admission.

^d^ *P* values are calculated using χ^2^ test or Kruskal-Wallis test.

In multivariable modeling, the interaction between the CRRT initiation category and VO was not significant (Wald χ^2^_1_ = 0.65; *P* = .42). The association of the CRRT initiation and VO categories was not associated with MAKE-90. Those with CRRT initiation at greater than 2 days and VO less than 10% had 1.7-fold greater adjusted odds of 90-day mortality compared with CRRT initiation at less than or equal to ≤2 days and VO less than 10% (aOR, 1.7 [95% CI, 1.2-2.4]; *P* = .006). Those with CRRT initiation at greater than or equal to 2 days and VO greater than or equal to 10% had 1.5-fold greater adjusted odds of 90-day mortality (aOR, 1.5 [95% CI, 1.1-2.2]; *P* = .03) compared with CRRT initiation at less than 2 days and VO less than 10%. There were no between-subphenotype differences in the odds of mortality (eFigure 5 in Supplement 1). Other associations with outcomes are summarized in eTable 4 in Supplement 1. Compared with CRRT initiation at less than or equal to 2 days and VO less than 10%, all other subphenotype groups had significantly fewer median ventilator and ICU-free days after adjusting for confounders (eFigure 6 in Supplement 1).

## Discussion

In this cohort study’s secondary analysis of the international multicenter WE-ROCK registry, we evaluated the association of timing of CRRT and VO at CRRT initiation with outcomes in a large pediatric cohort. Timing of CRRT initiation, anchored to ICU admission, was associated with worse outcomes. Longer time to CRRT initiation was associated with 3% greater odds of MAKE-90 for each 1 day later CRRT was initiated. There were 21% greater odds of MAKE-90 among those who initiated CRRT 6 days after ICU admission compared with those who initiated CRRT 1 day after ICU admission. Moreover, in an analysis of CRRT timing and VO together, 90-day mortality was higher in those with late initiation (>2 days), irrespective of VO.

The optimal timing of CRRT delivery in pediatric critical care remains unresolved. Recently, several studies have evaluated the timing of CRRT initiation and the association with outcomes.^11,31,32,33^ These studies reported that longer time to CRRT initiation was associated with worse outcomes.^31,32,33^ Our study found that the timing of CRRT initiation is associated with more than just mortality by using a composite outcome of MAKE-90. Mortality within 90 days was approximately 58% and among the survivors, approximately 44% had persistent kidney dysfunction, of which approximately 35% were dialysis dependent. These findings are particularly profound when one considers that the early CRRT initiation group was sicker, as measured by higher PRISM-III scores at ICU admission. This highlights the continued need to develop stratification tools aimed at identifying at-risk patients early in their ICU course.

The independent association of VO at CRRT initiation with adverse outcomes in children treated with CRRT was recognized more than 20 years ago.^39^ Since then, multiple studies have reported that higher VO at CRRT initiation is associated with adverse outcomes. Perhaps 1 of the most interesting findings in the current study is the median (IQR) VO of 7.4% (2.4%-18.1%) at CRRT initiation, which is lower than previously reported in the ppCRRT registry (median 9.6%).^7^ Furthermore, the bivariable comparisons in the current study showed no difference in VO at CRRT initiation and MAKE-90. This may reflect changing attitudes on CRRT initiation recently reported by independent clinician surveys performed by WE-ROCK,^17^ the European Society of Pediatric and Neonatal Intensive Care (ESPNIC)^16^ finding that percentage VO was increasingly used in CRRT initiation decisions.

Initiation of CRRT is determined by complex factors rather than thresholds of timing or VO alone; however, these factors may be key determinants of outcomes. Selewski and colleagues^50^ previously reported that percentage VO thresholds of greater than or equal to 5% and greater than or equal to 10% on ICU day 1 and 2 influenced patient-centered outcomes. In the current study, we investigated the interaction between VO and timing thresholds by performing a subphenotype analysis. Importantly, irrespective of the degree of percentage VO, there was an association of late CRRT initiation with 90-day mortality.

Our findings provide important information that must be interpreted in the global context of pediatric critical care nephrology research. An AKI clinical decision support algorithm using a sequential risk stratification tool (renal angina index), urine neutrophil gelatinase–associated lipocalin, and the furosemide stress test to optimize AKI prediction for early CRRT initiation in children who were critically ill was reported to have improved outcomes in a single center.^51^ The results will likely enhance our understanding of when to initiate CRRT and serve as a model to build similar tools for other populations. Indeed, the arbitrary nature of when to use CRRT extends to adults, as was highlighted by a recent review.^26^ The detrimental effects of an early initiation of CRRT in adults are not insignificant (increased risk for adverse events, longer term dialysis dependence, and greater health care utilization costs).^26^ Thus, any trial or prospective study evaluating CRRT timing in children will need to utilize sequential risk stratification and balance the potential for negative effects.

The current study also highlights some important points about high-risk populations for developing VO greater than or equal to 10%. In bivariable analysis, those who had VO of at least 10% at CRRT initiation were younger, more likely to have sepsis, and had higher severity of illness scores. Of these, patient age warrants special discussion. Overall, the higher degree of VO at CRRT initiation in the younger patients may be a surrogate for the complexity and challenges around performing CRRT in small children utilizing adult CRRT machines that are not approved for, but frequently used, in children who weigh less than 20 kg.^52^ It will be important to follow-up on whether younger patients experience more VO as CRRT devices designed for neonates and infants are increasingly utilized.

### Limitations

There are limitations to the current study, including the retrospective approach from which we can only establish associations, not causation. We also cannot rule out the potential for unmeasured and residual confounding to have resulted in bias in the reported exposure-outcome associations. In using the MAKE-90 outcome, we acknowledge that this outcome warrants further study in infants less than 1 year of age as this group has evolving kidney function. In the current study, the timing of AKI relative to ICU admission could not be determined, and the indication for CRRT start and AKI etiology were not collected, including whether this was a secondary complication after ICU admission. We acknowledge further study is necessary to identify the optimal method to define VO in children who are sick.^23^ As with many retrospective studies, missing data have to be taken into account. We did not include other modalities of renal replacement therapy. In addition, we predominantly included larger academic centers, with what we can assume are similar resources. Finally, we did not have representation from Central and South America, Africa, and Asia.

## Conclusions

In this cohort study of children and young adults, delayed initiation of CRRT was associated with MAKE-90 and increased resource utilization. This analysis of the large multinational WE-ROCK study begins to fill an important gap in the pediatric critical care nephrology literature by providing a detailed analysis of the association of CRRT initiation timing and VO with outcomes. Prospective multicenter studies are needed in children to delineate the appropriate time to initiate CRRT that optimizes survival and reduces long-term morbidity and health care utilization.

## Notes

Supplement 1. **eMethods.** Covariate Balancing Generalized Propensity Score (CBGPS) **eTable 1.** STROBE Checklist: Time to Continuous Renal Replacement Therapy Initiation and 90-Day Major Adverse Kidney Events in Children and Young Adults **eTable 2.** Demographics, Clinical Characteristics, and Outcomes of Patients With and Without Volume Overload at Initiation of Continuous Renal Replacement Therapy **eTable 3.** Interquartile Odds Ratio Estimation of Time to CRRT Initiation, Comparing Weighted Regression by Generalized Propensity Score (GPS) With the Outcome Model Method **eTable 4.** Multivariable Logistic Regression or Ordinal Regression Evaluating the Association of Time to Continuous Renal Replacement Therapy Initiation and Volume Overload Sub-Phenotypes With Outcomes **eFigure 1.** Sub-Phenotypes of Time to Continuous Renal Replacement Therapy Initiation and % Volume Overload **eFigure 2.** Covariate Balance Plot **eFigure 3**. Consort Flow Diagram **eFigure 4.** Kaplan-Meier Curver Summarizing the Probability of Receiving CRRT Over Time **eFigure 5.** Multivariable Logistic Regression Evaluating the Association Between Continuous Renal Replacement Therapy Initiation and Volume Overload Sub-Phenotypes and 90-Day Mortality **eFigure 6.** Predicted Median Ventilator and Intensive Care Unit Free Days Among the Continuous Renal Replacement Therapy Initiation and Volume Overload Phenotypes

Supplement 2.
Nonauthor Collaborators

Supplement 3.
Data Sharing Statement
