# Supplementary material for: Time to Continuous Renal Replacement Therapy Initiation and 90-Day Major Adverse Kidney Events in Children and Young Adults
Source: JAMA Netw Open. 2024 Jan 2;7(1):e2349871. doi: 10.1001/jamanetworkopen.2023.49871 (PMC10762580; doi:10.1001/jamanetworkopen.2023.49871)
Supplement: Supplement 1. — eMethods. Covariate Balancing Generalized Propensity Score (CBGPS) eTable 1. STROBE Checklist: Time to Continuous Renal Replacement Therapy Initiation and 90-Day Major Adverse Kidney Events in Children and Young Adults eTable 2. Demographics, Clinical Characteristics, and Outcomes of Patients With and Without Volume Overload at Initiation of Continuous Renal Replacement Therapy eTable 3. Interquartile Odds Ratio Estimation of Time to CRRT Initiation, Comparing Weighted Regression by Generalized Propensity Score (GPS) With the Outcome Model Method eTable 4. Multivariable Logistic Regression or Ordinal Regression Evaluating the Association of Time to Continuous Renal Replacement Therapy Initiation and Volume Overload Sub-Phenotypes With Outcomes eFigure 1. Sub-Phenotypes of Time to Continuous Renal Replacement Therapy Initiation and % Volume Overload eFigure 2. Covariate Balance Plot eFigure 3. Consort Flow Diagram eFigure 4. Kaplan-Meier Curver Summarizing the Probability of Receiving CRRT Over Time eFigure 5. Multivariable Logistic Regression Evaluating the Association Between Continuous Renal Replacement Therapy Initiation and Volume Overload Sub-Phenotypes and 90-Day Mortality eFigure 6. Predicted Median Ventilator and Intensive Care Unit Free Days Among the Continuous Renal Replacement Therapy Initiation and Volume Overload Phenotypes [file jamanetwopen-e2349871-s001.pdf]

Supplemental Online Content

Gist KM, Menon S, Anton-Martin P, et al; for the WE-ROCK Investigators. Time to continuous renal replacement therapy initiation and 90-day major adverse kidney events in children and young adults. *JAMA Netw Open.* 2024;7(1):e2349871. doi:10.1001/jamanetworkopen.2023.49871

eMethods. Covariate Balancing Generalized Propensity Score (CBGPS)

eTable 1. STROBE Checklist: Time to Continuous Renal Replacement Therapy Initiation and 90-Day Major Adverse Kidney Events in Children and Young Adults

eTable 2. Demographics, Clinical Characteristics, and Outcomes of Patients With and Without Volume Overload at Initiation of Continuous Renal Replacement Therapy

eTable 3. Interquartile Odds Ratio Estimation of Time to CRRT Initiation, Comparing Weighted Regression by Generalized Propensity Score (GPS) With the Outcome Model Method

eTable 4. Multivariable Logistic Regression or Ordinal Regression Evaluating the Association of Time to Continuous Renal Replacement Therapy Initiation and Volume Overload Sub-Phenotypes With Outcomes

eFigure 1. Sub-phenotypes of Time to Continuous Renal Replacement Therapy Initiation and % Volume Overload

eFigure 2. Covariate Balance Plot

eFigure 3. Consort Flow Diagram

eFigure 4. Kaplan-Meier Curve Summarizing the Probability of Receiving CRRT Over Time

eFigure 5. Multivariable Logistic Regression Evaluating the Association Between Continuous Renal Replacement Therapy Initiation and Volume Overload Sub-phenotypes and 90-day Mortality

eFigure 6. Predicted Median Ventilator and Intensive Care Unit Free Days Among the Continuous Renal Replacement Therapy Initiation and Volume Overload Phenotypes

This supplemental material has been provided by the authors to give readers additional information about their work.

© 2024 Gist KM et al. *JAMA Network Open.*

eMethods. Covariate Balancing Generalized Propensity Score (CBGPS)

The generalized propensity score weights were estimated using the CBGPS method, treating our exposure variable as continuous days to treatment. This method utilized a generalized method-of-moments framework, which ensured a set of moment conditions minimizing the association between the continuous exposure variable and baseline covariates as measured by their correlations when estimating the propensity score. Inverse probability of treatment weights (IPTW) were then computed for each participant to account for measured confounding. Univariate regression was used to estimate the treatment effect by regressing the outcome on the continuous exposure using a weighted regression model incorporating the CBGPS-based IPTW weights. The WeightIt package (version 0.13.1) in R (version 4.1.0) was used to generate the CBGPS and IPTW weights (https://ngreifer.github.io/WeightIt/reference/method_cbps.html). For additional detail on the CBGPS methodology we refer readers to "Fong, C., Hazlett, C., & Imai, K. (2018).[1]

Reference:

1. Fong C, Hazlett C, Imai K. Covariate balancing propensity score for a continuous treatment application to the efficacy of political advertisements. *The Annals of Applied Statistics*. 2018;12(1):156-177.

© 2024 Gist KM et al. *JAMA Network Open.*

eTable 1. STROBE Checklist: Time to Continuous Renal Replacement Therapy Initiation and 90-Day Major Adverse Kidney Events in Children and Young Adults

	Item No	Recommendation	Page
**Title and abstract**	1	(*a*) Indicate the study's design with a commonly used term in the title or the abstract	1
		(*b*) Provide in the abstract an informative and balanced summary of what was done and what was found	5
**Introduction**			
Background/rationale	2	Explain the scientific background and rationale for the investigation being reported	6
Objectives	3	State specific objectives, including any prespecified hypotheses	7
**Methods**			
Study design	4	Present key elements of study design early in the paper	8
Setting	5	Describe the setting, locations, and relevant dates, including periods of recruitment, exposure, follow-up, and data collection	8
Participants	6	(*a*) *Cohort study*—Give the eligibility criteria, and the sources and methods of selection of participants. Describe methods of follow-up	8
		(*b*) *Cohort study*—For matched studies, give matching criteria and number of exposed and unexposed	8, 9, eMethods
Variables	7	Clearly define all outcomes, exposures, predictors, potential confounders, and effect modifiers. Give diagnostic criteria, if applicable	9
Data sources/ measurement	8*	For each variable of interest, give sources of data and details of methods of assessment (measurement). Describe comparability of assessment methods if there is more than one group	*8, 9*
Bias	9	Describe any efforts to address potential sources of bias	10
Study size	10	Explain how the study size was arrived at	N/A
Quantitative variables	11	Explain how quantitative variables were handled in the analyses. If applicable, describe which groupings were chosen and why	9-11
Statistical methods	12	(*a*) Describe all statistical methods, including those used to control for confounding	9-11
		(*b*) Describe any methods used to examine subgroups and interactions	9-11
		(*c*) Explain how missing data were addressed	10-11
		(*d*) *Cohort study*—If applicable, explain how loss to follow-up was addressed	N/A
		€ Describe any sensitivity analyses	N/A

© 2024 Gist KM et al. *JAMA Network Open.*

eTable 2. Demographics, clinical characteristics, and outcomes of patients with and without volume overload at initiation of continuous renal replacement therapy.

Variable	Overall (n=975)	VO <10% (n=571)	VO ≥ 10% (n=404)	P-Value
**Age (years)**	8.8 (1.7-15.0)	11.7 (3.5-16.1)	3.7 (0.9-12.0)	<.001
**Admission weight (kg)**	26.8 (11.6-55.0)	42.0 (15.0-64.4)	16.0 (8.3-35.5	<.001
**Female**	444 (45.5)	260 (45.5)	184 (45.5)	>.99
**Male**	531 (54.4)	311 (54.4)	220 (54.4)	
**Race**				.30
**White**	56 (76.4)	384 (76.0)	272 (76.8)	
**Black**	126 (14.7)	68 (13.5)	58 (16.4)	
**Native American**	16 (1.9)	10 (2.0)	6 (1.7)	
**Asian/Pacific Islander**	43 (5.0)	31 (6.1)	12 (3.4)	
**More than 1 race**	18 (2.1)	12 (2.4)	6 (1.7)	
**Missing**	116	66	50	
**Ethnicity**				.003
**Non-Hispanic or Latino**	703 (81.6)	394 (78.2)	309 (86.3)	
**Hispanic or Latino**	159 (18.4)	110 (21.8)	49 (13.7)	
**Missing**	113	67	46	
**Admit Category**				<.001
**Shock/infection/Trauma**	364 (37.3)	179 (31.3)	185 (45.8)	
**Respiratory Failure**	194 (19.9)	116 (20.3)	78 (19.3)	
**Post-surgical/minor trauma**	49 (5.0)	22 (3.9)	27 (6.7)	
**CNS dysfunction**	39 (3.8)	22 (3.9)	9 (2.2)	
**Pain/sedation**	8 (0.8)	4 (0.7)	4 (1.0)	
**Primary Cardiac disease**	30 (3.2)	11 (1.9)	19 (4.7)	
**Post Cardiac Surgery**	47 (5.1)	21 (3.7)	26 (6.4)	
**Heart failure/myopathy**	39 (4.0)	32 (5.6)	7 (1.7)	
**Other**	205 (21.0)	156 (27.3)	49 (12.1)	
**Comorbidity**				
**None**	192 (19.7)	116 (20.3)	76 (18.8)	.67
**Respiratory**	131 (13.4)	67 (11.7)_	64 (15.8)	.08
**Cardiac**	190 (19.5)	93 (16.3)	97 (24.0)	.004
**Neurologic/neuromuscular**	131 (13.4)	64 (11.2)	67 (16.6)	.020
**Nephrologic/Urologic**	89 (9.1)	58 (10.1)	31 (7.7)	.22
**Hematologic**	132 (13.5)	84 (14.7)	48 (11.9)	.24
**Oncologic**	221 (22.7)	141 (24.7)	80 (19.8)	.09
**Immunologic**	153 (15.7)	98 (17.2)	55 (13.6)	.16
**Gastrointestinal**	184 (18.9)	97 (17.0)	87 (21.5)	.09
**Endocrinologic**	62 (6.4)	31 (5.4)	31 (7.7)	.20
**Comorbidities:**				..21
**None**	192 (19.7)	116 (20.3)	76 (18.8)	
**1**	478 (49.9)	283 (49.6)	195 (48.3)	
**2**	191 (19.6)	116 (20.3)	75 (18.6)	
**>2**	115 (11.8)	57 (10.0)	58 (14.3)	
**Baseline measured serum creatinine (mg/dL)**	0.4 (0.2-0.6) (n=537)	0.5 (0.3-0.7) (n=305)	0.3 (0.2-0.5) (n=232)	<.001
**Sepsis at ICU admission**	446 (45.7)	229 (40.1)	217 (53.7)	<.001
**PRISM-III at ICU Admission**	14 (10-18)	14 (10-18)	15 (10-19)	.079
**PELOD-2 CRRT Initiation**	7 (4-9)	6 (4-8)	7.5 (5-10)	<.001
**VIS at CRRT Initiation**	5 (0-20)	2 (0-14)	8 (0-23)	<.001
**Indexed UOP 24 hours prior to CRRT Initiation (ml/kg/hr)**	0.5 (0.1-1.2)	0.9 (0.5-1.1)	1.0 (0.6-1.7)	.001
**Time to CRRT Initiation (days)**	2 (1-6)	1 (0-4)	4 (2-10)	<.001

© 2024 Gist KM et al. *JAMA Network Open.*

**CRRT Duration (days)**	6 (3-14)	5 (3-13)	7 (4-15)	.003
**Ventilator Free days**	13 (0-28)	17 (0-28)	3 (0-28)	<.001
**ICU Free days**	0 (0-11)	0 (0-15)	0 (0-2)	<.001
**90-day Mortality**	366 (37.5)	202 (35.4)	164 (40.6)	.11
**MAKE-90**	626 (64.2)	361 (63.2)	265 (65.6)	.52

The overall sample is 975 (includes all patients with available fluid data). Categorical variables are presented as frequency with percent. Continuous variables are presented as median with interquartile range (IQR). P<0.05 denotes statistical significance. Abbreviations: MAKE-90 – major adverse kidney events at 90 days (persistent kidney dysfunction, dialysis dependence or death) kg - kilograms; CNS – central nervous system; ICU – intensive care unit; PRISM-III – pediatric risk of mortality score III; PELOD-2 – pediatric logistic organ dysfunction score; VIS – vasoactive inotrope score; VO – percent volume overload; UOP – urine output; ml/kg/hr – milliliters per kilogram per hour, CRRT – continuous renal replacement therapy.

© 2024 Gist KM et al. *JAMA Network Open.*

eTable 3. Interquartile odds ratio estimation of time to CRRT initiation, comparing weighted regression by generalized propensity score (GPS) with the outcome model method.

	MAKE-90	Mortality at 90 days	ICU-free days	Ventilator-free days
**Approach**	**OR (95% CI)**	**OR (95% CI)**	**OR (95% CI)**	**OR (95% CI)**
Outcome model estimate	1.14 (1.09-1.19)	1.18 (1.08-1.29)	0.27 (0.25-0.29)	0.79 (0.72-0.87)
GPS weighted estimate	1.21 (1.16-1.26)	1.20 (1.11-1.31)	0.31 (0.29-0.33)	0.78 (0.72-0.84)

© 2024 Gist KM et al. *JAMA Network Open.*

eTable 4. Multivariable logistic regression or ordinal regression evaluating the association of time to continuous renal replacement therapy initiation and volume overload sub-phenotypes with outcomes.

			MAKE-90	Mortality at 90 days	28-day Ventilator-free days	29-Day ICU Free days
**Variable**	**Reference**	**Contrast**	**OR (95% CI)**	**OR (95% CI)**	**OR (95% CI)**	**OR (95% CI)**
No comorbidity	No	Yes	0.5 (0.4-0.6)	0.6 (0.4-0.9)	1.0 (0.7-1.4)	1.6 (1.0-2.4)
Respiratory comorbidity	No	Yes	0.9 (0.6-1.4)	1.2 (0.7-1.9)	0.7 (0.5-1.1)	0.7 (0.5-1.1)
Cardiac comorbidity	No	Yes	1.51 (1.1-2.2)	1.5 (1.0-2.1)	0.7 (0.5-1.1)	0.6 (0.4-0.9)
Oncologic comorbidity	No	Yes	1.3 (0.9-1.9)	1.9 (1.4-2.5)	0.7 (0.5-0.9)	1.0 (0.7-1.4)
Immunologic comorbidity	No	Yes	1.9 (1.2-3.1)	2.2 (1.6-2.9)	0.4 (0.3-0.6)	0.5 (0.4-0.8)
Sepsis at ICU admission	No	Yes	1.12 (0.8-1.7)	1.4 (1.1-1.9)	0.6 (0.5-0.9)	0.7 (0.5-0.9)
VIS at CRRT initiation	0	20	1.1 (1.0-1.3)	1.3 (1.1-1.5)	0.7 (0.6-0.9)	0.6 (0.5-0.8)
CRRT duration (days)	3	14	1.5 (1.3-1.7)	1.0 (0.9-1.0)	0.9 (0.7-1.2)	0.3 (0.2-0.4)
Time to CRRT initiation category	≤2	>2 days	1.0 (0.7-1.4)	1.7 (1.2-2.4)	0.5 (0.4-0.8)	0.2 (0.2-0.3)
Volume overload category	< 10%	≥ 10%	0.9 (0.6-1.3)	1.3 (0.9-1.8)	0.8 (0.6-1.2)	0.6 (0.4-0.8)

Continuous variables (VIS at CRRT initiation and time to CRRT initiation) are presented as interquartile odds ratio with 95% confidence intervals. Multivariable logistic regression was used to assess associations MAKE-90 and 90-day mortality. Ordinal regression was used to assess associations with 28-day ventilator and ICU free days. All other categorical variables are presented as odds ratio with 95% CI). Abbreviations: MAKE-90 – major adverse kidney events at 90 days (persistent kidney dysfunction, dialysis dependence or death), ICU – intensive care unit, VIS = vasoactive inotrope score, CRRT = continuous renal replacement therapy.

© 2024 Gist KM et al. *JAMA Network Open.*

eFigure 1. Sub-phenotypes of time to continuous renal replacement therapy initiation and % volume overload. Four sub-phenotypes delineated by early (≤2 days) and late (≥2 days) continuous renal replacement therapy initiation, and % volume overload (<10% and ≥10%).

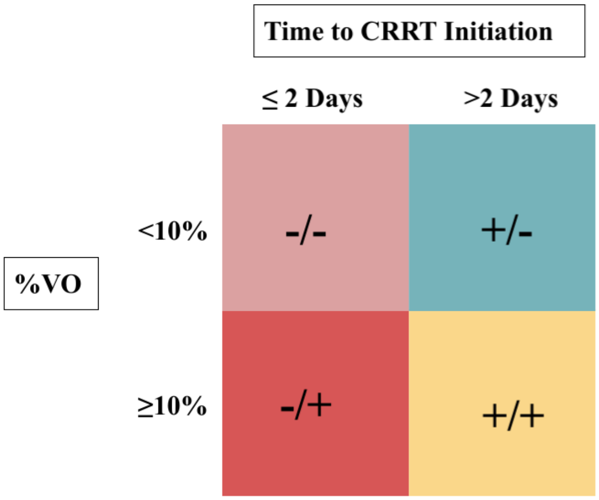

© 2024 Gist KM et al. *JAMA Network Open.*

eFigure 2. Covariate balance plot. The balance of each covariate was assessed using the correlation with the continuous exposure (i.e., time to CRRT initiation) before and after the covariate-balancing generalized propensity score (CBGPS) weighting. The missing covariate data (represented by NA) were handled by incorporating missingness in the CBGPS estimation process. The absolute correlation coefficients < 0.1 for most covariates suggest an overall good balance (i.e., minimal confounding) after weighting adjustment.

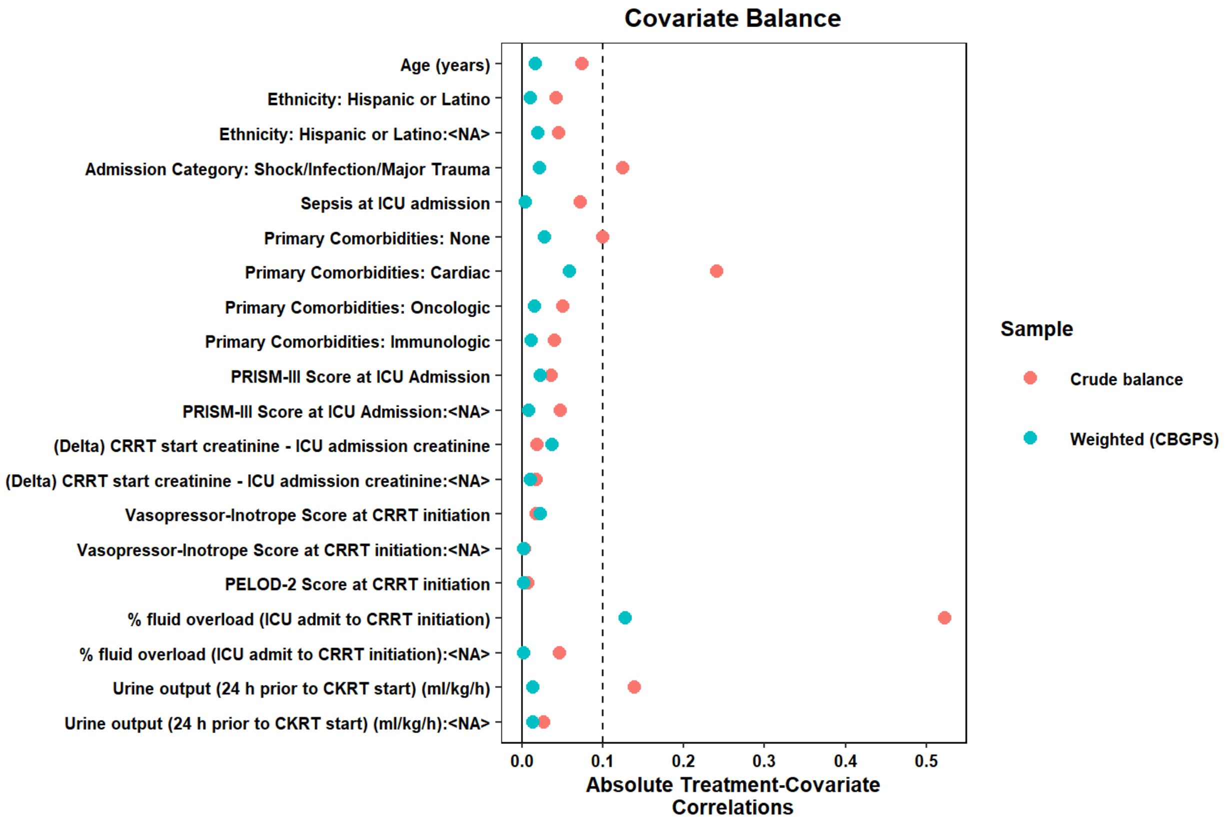

© 2024 Gist KM et al. *JAMA Network Open.*

eFigure 3. Consort flow diagram

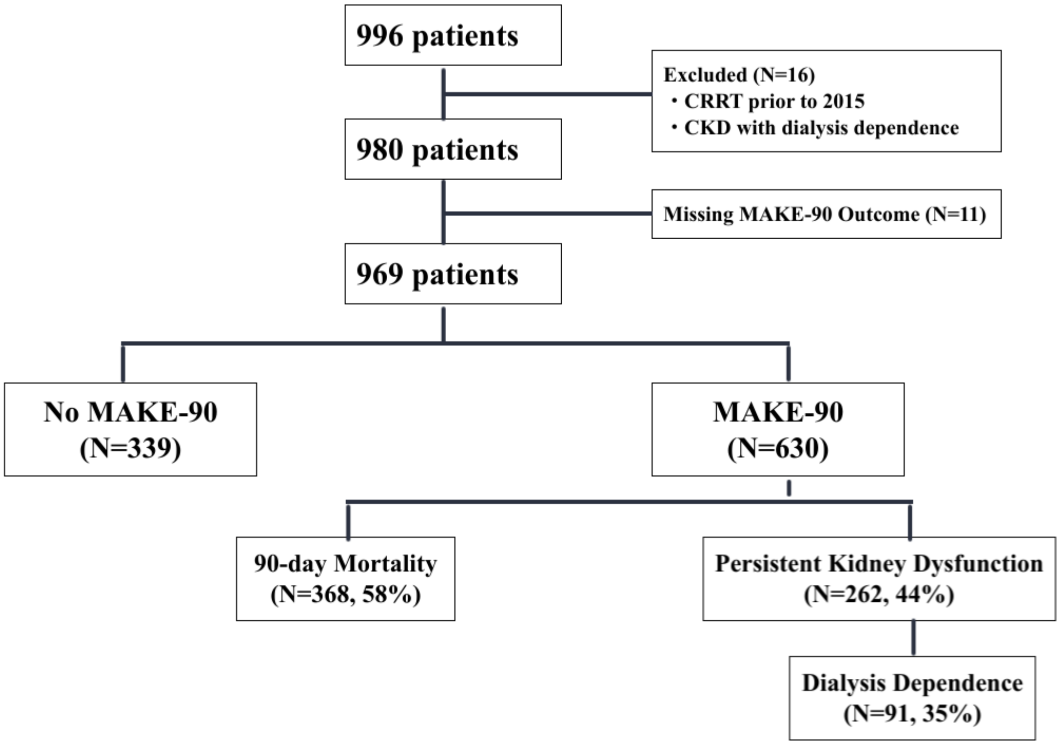

© 2024 Gist KM et al. *JAMA Network Open.*

eFigure 4. Kaplan-Meier Curve summarizing the probability of receiving CRRT over time. The probability of receiving CRRT increased within the first 5 days of ICU admission and then started to plateau. The number at risk, in intervals of every 5 days is summarized in the box below the x-axis.

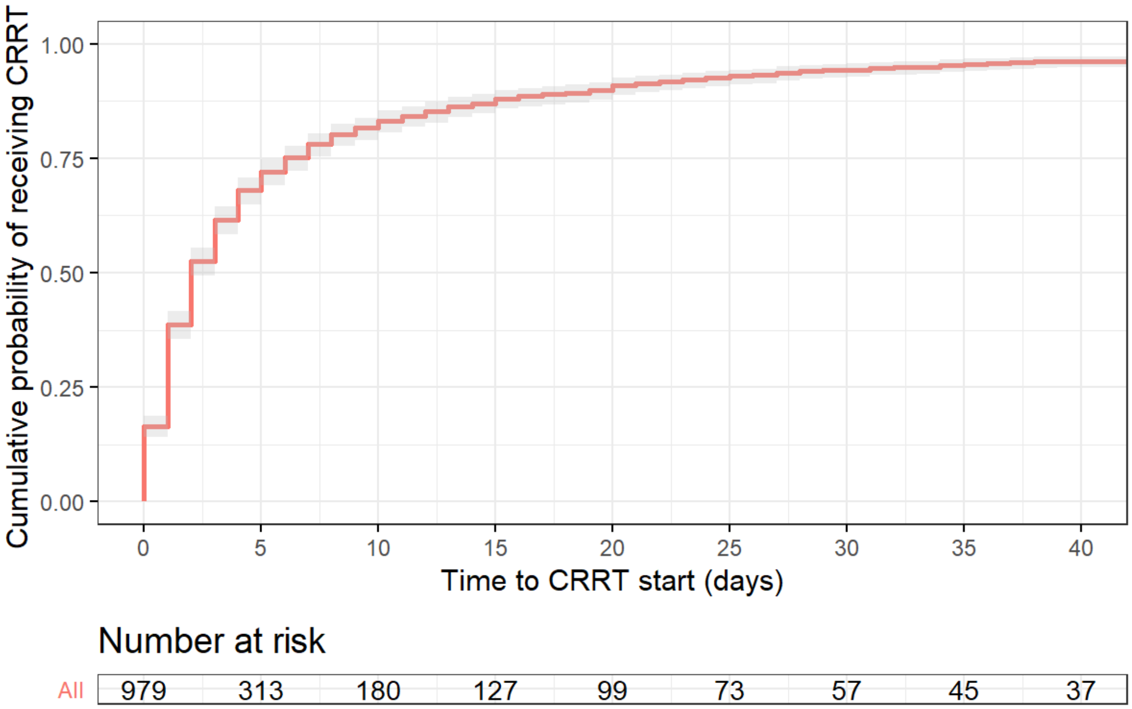

© 2024 Gist KM et al. *JAMA Network Open.*

eFigure 5. Multivariable logistic regression evaluating the association between continuous renal replacement therapy initiation and volume overload sub-phenotypes and 90-day mortality. Compared to the reference group, early initiation/<10 VO, both late CRRT/<10 VO and late CRRT/≥10 VO had a significantly greater odds of 90-day mortality. Early initiation was defined as ≤2 days and late initiation was defined as >2 days, both anchored to ICU admission. There were no significant between group differences in 90-day mortality after adjusting for confounders.

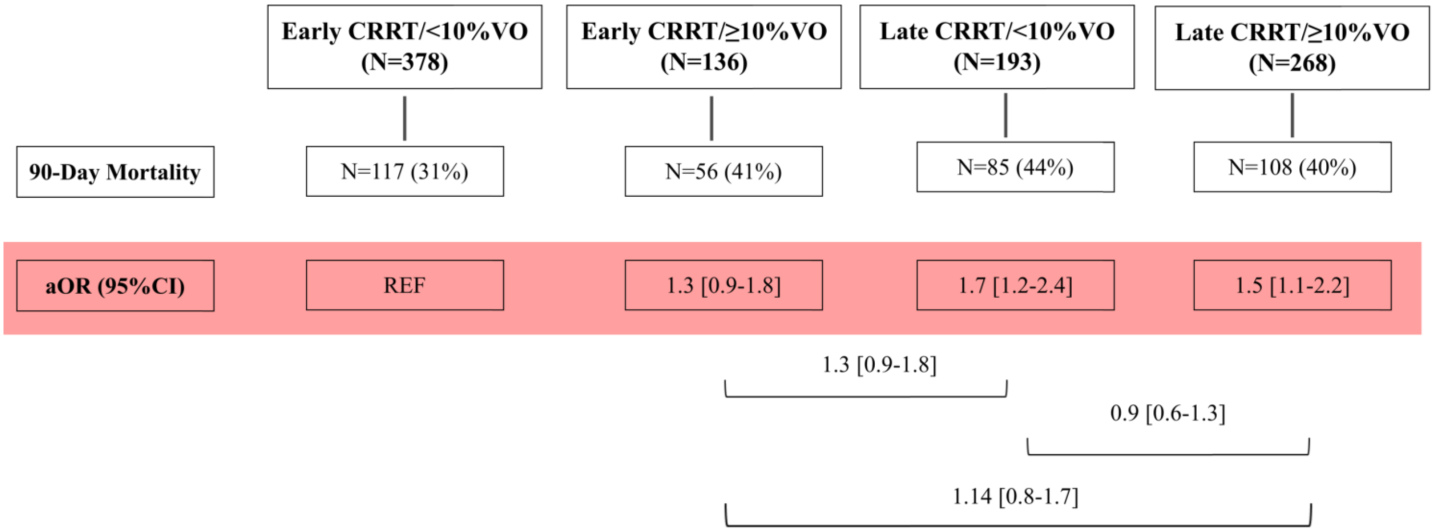

© 2024 Gist KM et al. *JAMA Network Open.*

eFigure 6. Predicted median ventilator and intensive care unit free days among the continuous renal replacement therapy initiation and volume overload phenotypes. Semiparametric ordinal regression models were used to calculate median ventilator (A) and ICU free days (B) across sub-phenotype groups. The models accounted for the nesting of patients within centers via the Huber–White cluster sandwich estimator of variance. The interaction between time to CRRT initiation (≤2 days vs >2 days) and %VO (<10% vs. ≥10%) was assessed and was not significant. The predicted median values were adjusted for no comorbidity, cardiac, respiratory, oncologic, immunologic comorbidities, sepsis at ICU admission and vasoactive inotrope score at CRRT initiation at the most frequent or median level.

A

	Early CRRT/<10%VO (N=378)	Early CRRT/≥10%VO (N=136)	Late CRRT/<10%VO (N=193)	Late CRRT/≥10%VO (N=268)
Ventilator Free Days (median, IQR)	21 (0-28)	11 (0-28)	2 (0-25)	0 (0-28)
Median (95%CI)	27.0 (21.5-27.2)	24.2 (18.2-27.2)	19.3 (11.9- 24.5)	18.6 (4.5- 25.7)
P-Value	REF	.3	<.001	<.001

B

	Early CRRT/<10%VO (N=378)	Early CRRT/≥10%VO (N=136)	Late CRRT/<10%VO (N=193)	Late CRRT/≥10%VO (N=268)
ICU Free Days (median, IQR)	6 (0-17)	0 (0-8)	0 (0-2)	0 (0-0)
Median (95%CI)	12.6 (10.2-15.0)	8.1 (1.5-12.4)	0 (0-1.8)	0 (0-0)
P-Value	REF	.005	<.001	<.001

© 2024 Gist KM et al. *JAMA Network Open.*
