# Supplementary material for: Time to Continuous Renal Replacement Therapy Initiation and 90-Day Major Adverse Kidney Events in Children and Young Adults
Source: JAMA Netw Open. 2024 Jan 2;7(1):e2349871. doi: 10.1001/jamanetworkopen.2023.49871 (PMC10762580; doi:10.1001/jamanetworkopen.2023.49871)
Supplement: Supplement 2. — Nonauthor Collaborators [file jamanetwopen-e2349871-s002.pdf]

Supplemental Online Content: Nonauthor Collaborators
*First name, last name, and suffix (if applicable) are required and will appear in PubMed.

*Group Name(s): WE-ROCK Investigators							
***First Name and Middle Initial(s)**	***Last Name**	***Suffix (eg, Jr, III)**	Academic Degrees	Institution	Location (city, state/province, country)	Role or Contribution, eg, chair, principal investigator	Group (if more than 1 Group listed in the byline) and/or Subgroup (eg, Steering Committee)
Emily	Ahern		CPNP, DNP	Children's Hospital Colorado, University of Colorado School of Medicine,	Aurora, CO, USA	Data collection, manuscript review	
Ayse	Akcan Arikan		MD	Baylor College of Medicine, Texas Children's Hospital	Houston, TX, USA	Data collection, manuscript review	
Issa	Alhamoud		MD	University of Iowa Stead Family Children's Hospital, Carver College of Medicine	Iowa City, IA, USA	Data collection, manuscript review	
Rashid	Alobaidi		MD	Univeristy of Alberta, Stollery Children's Hospital	Edmonton, Alberta, Canada	Data collection, manuscript review	
Shanthi S	Balani		MD	University of Minnesota	Minneapolis, MN, USA	Data collection, manuscript review	
Matthew	Barhight		MD, MS	Ann and Robert H. Lurie Children's Hospital of Chicago	Chicago IL, USA	Data collection, manuscript review	
Abby	Basalely		MD	Cohen Children's Medical Center, Zucker School of Medicine,	Hyde Park, NY, USA	Data collection, manuscript review	
Gabriella	Bottari		MD	Bambino Gesù Children’s Hospital, IRCCS	Rome, Italy	Data collection, manuscript review	
Andrea	Cappoli		MD	Bambino Gesù Children’s Hospital, IRCCS	Rome, Italy	Data collection, manuscript review	
Eileen A	Ciccia		MD	Washington University School of Medicine, St. Louis Children's	St Louis, MO, USA	Data collection, manuscript review	
Michaela	Collins		BA	Cincinnati Children's Hospital Medical Center; University of Cincinnati College of Medicine	Cincinnati OH, USA	Data collection, manuscript review	

*First name, last name, and suffix (if applicable) are required and will appear in PubMed.

***First Name and Middle Initial(s)**	***Last Name**	***Suffix (eg, Jr, III)**	Academic Degrees	Institution	Location (city, state/province, country)	Role or Contribution, eg, chair, principal investigator	Group (if more than 1 Group listed in the byline) and/or Subgroup (eg, Steering Committee)
Denise	Colosimo		MD	Meyer Children's Hospital, IRCCS	Florence, Italy	Data collection, manuscript review	
Mihaela A	Damian		MD, MPH	Stanford University School of Medicine	Palo Alto, CA, USA	Data collection, manuscript review	
Gabrielle	DeAbreu		MD	Cohen Children's Medical Center, Zucker School of Medicine	Hyde Park, NY, USA	Data collection, manuscript review	
Kathy L	Ding		BS	University of Colorado, School of Medicine	Aurora, CO, USA	Data collection, manuscript review	
Kristin J	Dolan		MD	Mercy Children's Hospital and Baylor College of Medicine, Texas Children's Hospital	Kansas City, MO and Houston, TX, USA	Data collection, manuscript review	
Sarah N	Fernandez LaFever		Md, PhD	Gregorio Marañón University Hospital; School of Medicine,	Madrid, Spain	Data collection, manuscript review	
Dana Y	Fuhrman		MD, MS	University of Pittsburgh Medical Center Children's Hospital of Pittsburgh	Pittsburgh, PA, USA	Data collection, manuscript review	
Francesco	Guzzi		MD	Santo Stefano Hospital	Prato, Italy	Data collection, manuscript review	
Taiki	Haga		MD	Osaka City General Hospital	Osaka, Japan	Data collection, manuscript review	
Elizabeth	Harvey		MD	Hospital for Sick Children	Toronto, Ontario, Canada	Data collection, manuscript review	
Denise C	Hasson		MD	Cincinnati Children's Hospital and NYU Langone Health, Hassenfeld Children’s Hospital,	Cincinnati OH, and New York, NY, USA	Data collection, manuscript review	
Taylor	Hill-Horowitza		BS	Cohen Children's Medical Center, Zucker School of Medicine,	Hyde Park, NY, USA	Data collection, manuscript review	
Haleigh	Inthavong		BS, MS	Baylor College of Medicine, Texas Children's Hospital	Houston, TX, USA	Data collection, manuscript review	

Supplemental Online Content: Nonauthor Collaborators
*First name, last name, and suffix (if applicable) are required and will appear in PubMed.

***First Name and Middle Initial(s)**	***Last Name**	***Suffix (eg, Jr, III)**	Academic Degrees	Institution	Location (city, state/province, country)	Role or Contribution, eg, chair, principal investigator	Group (if more than 1 Group listed in the byline) and/or Subgroup (eg, Steering Committee)
Catherine	Joseph		MD	Baylor College of Medicine, Texas Children's Hospital	Houston, TX, USA	Data collection, manuscript review	
Ahmad	Kaddourah		MD, MS	Sidra Medicine and Weil Cornel Medicine	Doha, Qatar	Data collection, manuscript review	
Aadil	Kakajiwala		MD	Children's National Hospital	Washington DC, USA	Data collection, manuscript review	
Aaron D	Kessel		MD, MS	Cohen Children's Medical Center, Zucker School of Medicine,	Hyde Park, NY, USA	Data collection, manuscript review	
Sarah	Korn		DO	Westchester Medical Center	Westchester, NY, USA	Data collection, manuscript review	
Kelli A	Krallman		BSN, MS	Cincinnati Children's Hospital Medical Center; University of Cincinnati College of Medicine	Cincinnati OH, USA	Data collection, manuscript review	
David M	Kwiatkowski		MD, MSc	Stanford University School of Medicine	Palo Alto, CA, USA	Data collection, manuscript review	
Jasmine	Lee		MSc	Hospital for Sick Children	Toronto, Ontario, Canada	Data collection, manuscript review	
Laurance	Lequier		MD	Univeristy of Alberta, Stollery Children's Hospital	Edmonton, Alberta, Canada	Data collection, manuscript review	
Tina	Madani Kia		BS	Univeristy of Alberta, Stollery Children's Hospital	Edmonton, Alberta, Canada	Data collection, manuscript review	
Eleonora	Marinari		MD	Bambino Gesù Children’s Hospital, IRCCS	Rome, Italy	Data collection, manuscript review	
Susan D	Martin		MD	Golisano Children's Hospital at University of Rochester Medical Center	Rochester, NY, USA	Data collection, manuscript review	

Supplemental Online Content: Nonauthor Collaborators
*First name, last name, and suffix (if applicable) are required and will appear in PubMed.

***First Name and Middle Initial(s)**	***Last Name**	***Suffix (eg, Jr, III)**	Academic Degrees	Institution	Location (city, state/province, country)	Role or Contribution, eg, chair, principal investigator	Group (if more than 1 Group listed in the byline) and/or Subgroup (eg, Steering Committee)
Tahagod H	Mohamed		MD	Nationwide Children's Hospital, The Ohio State University College of Medicine	Columbus, OH, USA	Data collection, manuscript review	
Catherine	Morgan		MD	Univeristy of Alberta, Stollery Children's Hospital	Edmonton, Alberta, Canada	Data collection, manuscript review	
Theresa	Mottes		APRN	Ann and Robert H. Lurie Children's Hospital of Chicago	Chicago IL, USA	Data collection, manuscript review	
Melissa A	Muff-Luett		MD	University of Nebraska Medical Center, Children's Hospital & Medical Center,	Omaha, NE, USA	Data collection, manuscript review	
Siva	Namachivayam		MBBS	Royal Children's Hospital, University of Melbourne, Murdoch Children's Research Institute	Melbourne, Victoria, Australia	Data collection, manuscript review	
Tara M	Neumayr		MD	Washington University School of Medicine, St. Louis Children's	St Louis, MO, USA	Data collection, manuscript review	
Jennifer	Nhan		MD	Children's National Hospital	Washington DC, USA	Data collection, manuscript review	
Abigail	O'Rourke		MD	Cohen Children's Medical Center, Zucker School of Medicine,	Hyde Park, NY, USA	Data collection, manuscript review	
Matthew G	Pinto		MD	Maria Fareri Children's Hospital at Westchester Medical Center,	Westchester, NY, USA	Data collection, manuscript review	
Dua	Qutob		MD	Sidra Medicine and Weil Cornel Medicine	Doha, Qatar	Data collection, manuscript review	
Valeria	Raggi		MD	Bambino Gesù Children’s Hospital, IRCCS	Rome, Italy	Data collection, manuscript review	

*First name, last name, and suffix (if applicable) are required and will appear in PubMed.

***First Name and Middle Initial(s)**	***Last Name**	***Suffix (eg, Jr, III)**	Academic Degrees	Institution	Location (city, state/province, country)	Role or Contribution, eg, chair, principal investigator	Group (if more than 1 Group listed in the byline) and/or Subgroup (eg, Steering Committee)
Stephanie	Reynaud		MD	Hospital for Sick Children and Hopital Bicetre, APHP Université Paris-Saclay	Toronto, Ontario, Canada and Kremlin-Bicetre, Val de Marne, France	Data collection, manuscript review	
Zaccaria	Ricci		MD	Meyer Children's Hospital, IRCCS	Florence, Italy	Data collection, manuscript review	
Zachary A	Rumlow		DO	University of Iowa Stead Family Children's Hospital, Carver College of Medicine	Iowa City, IA, USA	Data collection, manuscript review	
María J	Santiago Lozano		MD, PhD	Gregorio Marañón University Hospital; School of Medicine	Madrid, Spain	Data collection, manuscript review	
Emily	See		MBBS, PhD	Royal Children's Hospital, University of Melbourne, Murdoch Children's Research Institute	Melbourne, Victoria, Australia	Data collection, manuscript review	
Carmela	Serpe		PhD	Bambino Gesù Children’s Hospital, IRCCS	Rome, Italy	Data collection, manuscript review	
Alyssa	Serratore		RN, MSc	Royal Children's Hospital, University of Melbourne, Murdoch Children's Research Institute	Melbourne, Victoria, Australia	Data collection, manuscript review	
Ananya	Shah		BS	University of Colorado, School of Medicine	Aurora, CO, USA	Data collection, manuscript review	
Weiwen V	Shih		MD	University of Colorado, School of Medicine	Aurora, CO, USA	Data collection, manuscript review	
Cara L	Slagle		MD	Cincinnati Children's Hospital Medical Center; University of Cincinnati College of Medicine	Cincinnati OH, USA	Data collection, manuscript review	
Sonia	Solomon		DO	Westchester Medical Center, Westchester	Westchester, NY, USA	Data collection, manuscript review	

Supplemental Online Content: Nonauthor Collaborators
*First name, last name, and suffix (if applicable) are required and will appear in PubMed.

***First Name and Middle Initial(s)**	***Last Name**	***Suffix (eg, Jr, III)**	Academic Degrees	Institution	Location (city, state/province, country)	Role or Contribution, eg, chair, principal investigator	Group (if more than 1 Group listed in the byline) and/or Subgroup (eg, Steering Committee)
Danielle E	Soranno		MD	Indiana University School of Medicine, Riley Hospital for Children	Indianapolis, IA, USA	Data collection, manuscript review	
Rachana	Srivastava		MD	Mattel Children's Hospital at UCLA	Los Angeles, CA, USA	Data collection, manuscript review	
Natalja L	Stanski		MD	Cincinnati Children's Hospital Medical Center; University of Cincinnati College of Medicine	Cincinnati OH, USA	Data collection, manuscript review	
Michelle C	Starr		MD, MPH	Indiana University School of Medicine, Riley Hospital for Children	Indianapolis, IA, USA	Data collection, manuscript review	
Erin K	Stenson		MD	University of Colorado, School of Medicine	Aurora, CO, USA	Data collection, manuscript review	
Amy E	Strong		MD, MSCE	University of Iowa Stead Family Children's Hospital, Carver College of Medicine	Iowa City, IA, USA	Data collection, manuscript review	
Susan A	Taylor		MSc	King's College Hospital	London, England, United Kingdom	Data collection, manuscript review	
Brynna	Van Wyk		ARNP, MSN	University of Iowa Stead Family Children's Hospital, Carver College of Medicine	Iowa City, IA, USA	Data collection, manuscript review	
Tennille N	Webb		MD, MSPH	Children's of Alabama/University of Alabama at Birmingham	Birmingham, AL, USA	Data collection, manuscript review	
Emily E	Zangla		DO	University of Minnesota	Minneapolis, MN, USA	Data collection, manuscript review	
