# Supplementary material for: Time to Continuous Renal Replacement Therapy Initiation and 90-Day Major Adverse Kidney Events in Children and Young Adults
Source: JAMA Netw Open. 2024 Jan 2;7(1):e2349871. doi: 10.1001/jamanetworkopen.2023.49871 (PMC10762580; doi:10.1001/jamanetworkopen.2023.49871)
Supplement: Supplement 3. — Data Sharing Statement [file jamanetwopen-e2349871-s003.pdf]

Data Sharing Statement

Gist. Time to Continuous Renal Replacement Therapy Initiation and 90-Day Major Adverse Kidney Events in Children and Young Adults. *JAMA Netw Open*. Published January 02, 2024. doi:10.1001/jamanetworkopen.2023.49871

Data
Data available: No

Additional Information
Explanation for why data not available: This data is part of an existing registry. Requests for ancillary studies can be made at the https://www.werockstudy.org/
